# Awareness of health risks related to body art practices among youth in Naples, Italy: a descriptive convenience sample study

**DOI:** 10.1186/1471-2458-11-625

**Published:** 2011-08-05

**Authors:** Francesca Gallè, Caterina Mancusi, Valeria Di Onofrio, Aniello Visciano, Vincenza Alfano, Roberto Mastronuzzi, Marco Guida, Giorgio Liguori

**Affiliations:** 1 Department of Studies of Institutions and Territorial Systems, University "Parthenope", Naples, Italy; 2 Department of Biological Sciences, Section of Physiology and Hygiene, University "Federico II", Naples, Italy

**Keywords:** Tattooing, Body Piercing, Risk Factors

## Abstract

**Background:**

Body art practices have emerged as common activities among youth, yet few studies have investigated awareness in different age groups of possible health complications associated with piercing and tattooing.

**Methods:**

We investigated perceptions of and knowledge about health risks. To highlight differences among age groups, we gathered data from students at high schools and universities in the province of Naples.

**Results:**

Of 9,322 adolescents, 31.3% were pierced and 11.3% were tattooed. Of 3,610 undergraduates, 33% were pierced and 24.5% were tattooed (p < 0.05). A higher number of females were pierced in both samples, but there were no gender differences among tattooed students. Among high school students, 79.4% knew about infectious risks and 46% about non-infectious risks; the respective numbers among university students were 87.2% and 59.1%. Only 3.5% of students in high school and 15% of university undergraduates acknowledged the risk of viral disease transmission; 2% and 3% knew about allergic risks. Among adolescents and young adults, 6.9% and 15.3%, respectively, provided signed informed consent; the former were less knowledgeable about health risks (24.7% vs. 57.1%) (p < 0.05). Seventy-three percent of the high school students and 33.5% of the university students had body art done at unauthorized facilities. Approximately 7% of both samples reported complications from their purchased body art.

**Conclusions:**

Results indicate a need for adequate information on health risks associated with body art among students in Naples, mainly among high school students. Therefore, adolescents should be targeted for public health education programs.

## Background

Recently, piercing and tattooing have gained increasing popularity worldwide. Although the literature differs on the basis of area and population studied, it indicates that body art is increasingly accepted by all social classes and age groups, but especially by youths [1-3]. In Western society, body piercing and tattooing have become mainstream activities among adolescents (12 to 18 years of age) and young adults (18 to 25 years of age) [1,3,4]. Prevalence of body art in these age groups vary by country and setting, ranging from 6.5% to 56% for pierced subjects, and from 4.5% to 24% for tattooed [5-13].

In Italy, three studies have been carried out in adolescents in Tuscany and the Veneto region. Data show that 20% to 35% of adolescents reported having a piercing, while 4% to 6.3% of students had tattoos [14-17].

As the prevalence of body art has increased, adverse health risks associated with these practices have been documented. The use of needles and other piercing instruments allows the mucocutaneous transmission of infectious diseases. These can range from local to systemic infections (e.g., osteomyelitis, toxic shock syndrome, and bacteremia), as well as to life-threatening ones (e.g., septic arthritis, acute glomerulonephritis, endocarditis, and hepatitis). In addition, the introduction of materials such as pigments and metals under the skin involve noninfective risks, such as allergic reactions. Later infectious complications brought on by lack of proper care of tattooed/pierced sites are also possible [1-3,18].

Given the widespread practice of tattooing and piercing, and the high number of potentially associated complications, it is important to inform consumers about possible hazards and the management of complications during and after these practices. However, youth often lack awareness of health risks, and patronize operators who ignore or do not apply risk-control measures [3].

In 1998, the Italian Ministry of Health issued safe practice guidelines that apply to tattoo and piercing facilities, as well as beauty shops. Many regions have since adopted these and established local regulations, and many professional piercers and tattooists have organized in association with health workers to promote safe body modification. Nonetheless, unlicensed and untrained personnel practicing body art out of unregulated shops, jewelry stores or at home can still provide youths with ready access to these services.

The majority of surveys on youths' knowledge of risks related to body art practices have been carried out among university students; those that address adolescents' attitudes are infrequent. In addition, these studies typically focus on perceptions about either tattoos or piercing, but rarely both [10,19,20]. No comparative studies on the body art practices, attitudes, and behaviors of both adolescents and young adults have been performed. To highlight possible differences among age groups, the present study investigated experiences with and knowledge of possible health consequences of tattooing and piercing by comparing two samples of students, those from high schools and universities in the province of Naples.

This research is part of a multidisciplinary project developed by the Chair of Hygiene of the University of Naples "Parthenope" and the Local Health Authority of the Campania region to introduce regional guidelines and educate body art workers and their customers about possible health risks and needed safety practices. This investigation focused on identification of the best population to target for further health information campaigns. We hypothesized that adolescents would meet that criterion.

## Methods

We administered a questionnaire to high school and college students in the province of Naples during the 2008-2009 academic year (Additional file 1). Two age group samples were involved in the study. The first was made up of students at 23 public secondary schools, while the second consisted of undergraduate students at seven universities. Both groups were convenience samples selected on the basis of teacher agreement to take part in the study. Included schools represented all of the six courses of studies of Italian public secondary schools (i.e., arts, humanities, sciences, technologies, economics, and vocational training). The university level was comprised of technical colleges and the representative disciplines of biomedicine, business, and the humanities. To avoid confounding by education on hygiene, only undergraduates at the start of their first academic year were included in the study.

We used a structured anonymous questionnaire that prompted yes/no or multiple choice answers to collect data on demographics, knowledge of health risks, and personal experience with tattooing and body piercing. The first section of the questionnaire focused on gender, age, and province of residence. In the second section, information on awareness about infectious and non-infectious risks associated with tattoos and piercing, and the ways to eliminate them was gathered. The third section was designed to identify adolescents who were tattooed or pierced and those interested in undergoing these procedures. Those who had at least one tattoo or piercing were asked to explain when, why, where, and under what conditions they had the body modification, and to report possible complications. Amateur tattooing/body piercing was not considered.

The questionnaire was refined after testing on a convenience sample of 100 university students. Since it was anonymous and self-completed, parental consent for participants < 18 years of age was waived. At the time of the interview, a researcher explained the purpose of the study and emphasized the anonymity of the responses. Ethical approval was obtained from the Local Education Office for the Campania Region and from the academic deans.

### Analysis

We used the chi-square test with Yates correction for comparisons between the two age groups, and the F-test for comparisons of the mean age at the first body modification. A p value < 0.05 was considered statistically significant. All analyses were performed using SPSS version 15.0 (IBM SPSS, Chicago, IL, USA).

## Results

The first sample (high school) included 9,322 students; the second, 3,610 university undergraduates. All participants returned the questionnaires, even if they did not always answer every question. Table 1 shows the demographic data of the two age groups, together with the number and percentage of pierced and tattooed youths, their age at first body art experience, and the number of students willing to try a piercing or a tattoo.

**Table 1:** Characteristics of the two student samples

	HIGH SCHOOLS n.: 9,322	UNIVERSITIES n.: 3,610	statistic	p value
Age (mean ± SD)	16.1 ± 1.3	21.6 ± 4.1		
Gender (%)*	4,557 (48.9) M 4,737 (50.8) F	2,052 (56.8) M 1,542 (42.7) F	χ^2 ^= 67.45	< 0.0000001
Pierced (%)	2,923 (31.3)	1,192 (33)	χ^2 ^= 3.319	0.06849
Gender of pierced (%)*	910 M (31.1) 2,008 F (68.6)	376 M (31.5) 811 F (68.0)	χ^2 ^= 0.09446	0.7586
Age at first piercing (mean ± SD)	12.9 ± 2.5	15.9 ± 4.3	F = 2.95	< 0.0000001
Tattooed (%)	1,054 (11.3)	886 (24.5)	χ^2 ^= 357.6	< 0.0000001
Gender of tattooed (%)*	534 M (50.6) 520 F (49.3)	311 M (35.1) 320 F (36.1)	χ^2 ^= 1.086	0.2991
Age at first tattoo (mean ± SD)	14.8 ± 2.5	17.6 ± 2.6	F = 2.95	0.2228
Interested to piercings	1,638 (25.5)	352 (14.5)	χ^2 ^= 122.2	< 0.0000001
Interested to tattooing	3,522 (42.5)	690 (25.3)	χ^2 ^= 412.9	< 0.0000001

*: some interviewed did not respond.

The two samples differed significantly in gender distribution (p < 0.0000001), with fewer females in the university group and more pierced females overall. We also found significant differences between the proportion of tattooed students in both groups (p < 0.0000001), but with an equal number of male and female students (p = 0.7586 and p = 0.2991).

Compared with university undergraduates, a significantly greater number of high school students were interested in getting a piercing or tattoo (25.5 vs. 14.5%, respectively, for piercing and 42.5 vs. 25.3%, respectively, for tattooing). Adolescents received their first piercing or tattoo at an earlier age than college students, with a mean age of 13 years for piercing and 15 years for tattooing. The respective figures for undergraduates were approximately 16 and 18 years of age. The only significant difference between the two samples was for piercing (p < 0.0000001).

Approximately 79% of high school students and 87% of undergraduates knew about the possible transmission of infectious diseases through body art practices; some 3.5% of the former and 15% of the latter identified hepatitis B and C viruses and HIV among the transmittable agents. Among adolescents, 46% associated non-infectious risks with piercing and tattooing; at the university level, the number was 59%. However, only 2% of high school students and 3% of university students considered the development of allergies, bleeding and cysts as potentially associated risks (Table 2).

**Table 2:** Number (%) of high school and university students who knew possible health risks of body art practices

	HIGH SCHOOLS n.: 9,322	UNIVERSITIES n.: 3,610	χ^2^statistic	p value
Infectious diseases (%)	7,404 (79.4)	3,148 (87.2)	104.3	< 0.0000001

Hepatitis and HIV (%)	259 (3.5)	472 (14.9)	450.9	< 0.0000001

Non-infectious diseases (%)	4,292 (46)	2,134 (59.1)	510.3	< 0.0000001

Allergies, bleeding, and cysts (%)	86 (2)	64 (2.9)	5765	0.016

A majority of students recognized surgical intervention for tattoos and spontaneous closing for piercings, as a means of removal. In the first sample, the respective figures were 59% and 74%; in the second, the figures were approximately 56% and 72% (p < 0.0000001) (data not shown).

The last section of the questionnaire gathered data from subjects with at least one tattoo or piercing (3,433 and 1,600 for the two groups, respectively). They were asked about motivation, information received, the place where the procedure was carried out, and about complications.

The most frequent motivation among high schools students who obtained a tattoo or a piercing was "it is fashionable" (25.7%), while university students followed the majority, "I do not know why" (22.4%). Parents were informed about the body modification by 2,510 (73.1%) of the adolescents and by 950 (59.3%) of the undergraduates.

Table 3 shows results regarding the receipt of health risk information. Compared with university students, a lower proportion of high school students provided signed informed consent at the time of the body art purchase (6.9% vs. 15.3%, p < 0.0000001). Moreover, adolescents were also less informed than undergraduates about health risks (24.7% vs. 57.1%, p < 0.0000001). The source of information did not differ significantly between the two groups (p = 0.2293); for both, the main source was a conversation with the tattooist or piercer.

**Table 3:** Number (%) of tattooed and pierced youth belonging to the two groups who signed an informed consent and received information about health risks; for the latest group, the source of information is reported

	HIGH SCHOOLS n.: 3,433	UNIVERSITIES n.: 1,600	χ^2^statistic	p value
Informed consent signed (%)	239 (6.9)	245 (15.3)	87.56	< 0.0000001

Health risks information received (%)	848 (24.7)	914 (57.1)	504.3	< 0.0000001
- through informed consent	185 (21.8)	223 (24.4)		
- from the operator	377 (44.4)	414 (45.3)	2.946	0.2293
- from another source	286 (33.7)	277 (30.3)		

Table 4 shows the number (%) of tattooed and pierced youths who patronized licensed facilities; the proportion of those who observed the use of sterile/disposable instruments during the procedure; and the number (%) of those who reported complications.

**Table 4:** Numbers (%) of tattooed/pierced students belonging to the two groups who attended licensed facilities (a beautician or a licensed tattooist/piercer practice), observed the use of sterile/disposable instruments and reported complications (infectious or noninfectious)

	HIGH SCHOOLS n.: 3,433	UNIVERSITIES n.: 1,600	χ^2^statistic	p value
Addressed to authorized facility (%)	927 (27.0)	1064 (66.5)	836.9	< 0.0000001
- beautician	276 (8)	206 (12.8)		
- tattooist/piercer	651 (18.9)	858 (53.6)		

Sterile/disposable instruments (%)	960 (27.9)	1,126 (70.3)	808.9	< 0.0000001

Complications suffered (%)	234 (6.8)	117 (7.3)	0.4144	0.5198
- infectious	176 (75.2)	22 (18.8)		
- non-infectious	58 (24.7)	95 (81.1)	100.9	< 0.0000001

Only 27% of tattooed and pierced high school students went to an authorized operator. The proportion was higher for undergraduates (66.5%), with a significant difference between the two groups (p < 0.0000001). A higher proportion of university students (70.3%) also observed the use of sterile/disposable instruments compared to high school students (27.9%). This difference was statistically significant (p < 0.0000001). Approximately 7% of both adolescents and undergraduates reported complications from their body art (p = 0.5198). Infections were more common among high school than university students, who mainly reported non-infectious complications (p < 0.0000001).

## Discussion

This study indicates that high school students have more body art than their university peers and less knowledge of the health risks associated with tattoos and piercings. They buy these products at a younger age, and greater numbers of them intend to obtain a tattoo or piercing or were interested in getting one.

In recent years, the number of young adults who purchase body art has increased. This is probably due to the growing use of body modification by media celebrities, sports icons, and peers [13,21]. However, this trend is accompanied by an increase in reported health complications, which are often due to limited knowledge by consumers about possible negative consequences of tattoos and body piercings.

To better characterize awareness of these risks among young populations who get these products, and to highlight possible differences among age groups, we surveyed secondary and university students in the province of Naples. They were asked to respond to a questionnaire on their knowledge of health risks and their personal experiences with tattoos and piercings. The return rate of 100% was most likely related to the presence of teachers while students filled out the questionnaires.

Our outcomes differed from those of other surveys conducted among high school students in Italy [14-16]. Boncompagni et al. found similar numbers of pierced adolescents (35% vs. 31% in this study) compared with 20% in Cegolon et al. and Bosello et al. Similarly the proportion of tattooed high school students in Naples (about 11%) was higher than that reported in both Tuscany and the Veneto regions (4.0% to 6.3%). The number of adolescents who were interested in piercing was lower or quite similar to prior Italian studies (23.5% vs. 25-43%), but fewer intended to get a tattoo (42.5% vs. 47-62%). This could be due to the higher proportion of already-tattooed youths in our sample.

More females were pierced in both groups, as reported in other studies [5,6,13,14]. The number of males and females among tattooed youths was similar for each age group, but a significant number of undergraduates did not declare their gender. A significant number of students in both groups claimed to be aware of infectious and non-infectious risks associated with tattoos and body piercings, but these percentages decreased when they were asked to identify viral diseases and allergic reactions possibly associated with these practices. This suggests an inadequate level of information. Although **around **60% of each group identified surgery as the mode of removal for tattoos, it has to be noted that the lack of a specific response about laser among those provided might have been confusing. However, many of the students indicated "laser" as another method in the open answer provided.

Knowledge of health risks was higher among undergraduates than adolescents. The increase of risk awareness with age was already showed by Cegolon et al. [15,16]. However, more secondary school students were aware of correct modes of elimination of tattoos and piercings than their older colleagues. Motivations to have body art varied. The majority of high school students did it because "it is fashionable," while the most frequent response among university students was "I don't know why."

Armstrong reported that common motivations among high school students were "it is a form of body art, it is fashionable, it makes a personal statement, and it is daring" [4]. More recently reasons for body piercing among college students are "uniqueness" and "to be myself" [7].

A 1997 survey by Armstrong and Pace Murphy found that 44% of adolescents "just wanted one" tattoo, 23% wanted to be independent and express themselves, and 16% did it "for the heck of it" [9]. A later report in 196 subjects who presented for tattoo removal [22] found that the major reasons for getting a tattoo were "helped me feel unique" and "helped me feel independent". On the basis of a literature analysis, Wohlrab et al. reported the expression of individuality and body embellishment as main motivations [23]. Our results are consistent with these findings among adolescents. However, the majority of undergraduates had other motivations that most likely reflected their desire to try body modification and see if they liked it.

More adolescents told their parents about their body art purchases than undergraduates. However, only 6.9% of high school students or their parents provided signed informed consent. The high percentage (76%) of adolescents who attended unauthorized facilities is consistent with these data, as is the mean age of < 18 years for first-time piercings and tattoos. This point is particularly pertinent in that Italy prohibits the practice of body art on underage adolescents without parental approval. It seems that adolescents who want body art will find a way to obtain it, regardless of cost, risks, or parental non-support [24]. Therefore, discussion leading to informed decision-making rather prohibition appears to be a sounder strategy.

While the personal experiences of adolescents indicate that they are less informed than undergraduates about health risks, the studio artist was the primary source of information for both groups. This suggests that tattooists and piercers can be effective sources of information about health risks.

A greater number of university students observed the use of sterile or disposable instruments, most likely due to their higher awareness of the role of these devices in the transmission of infectious diseases. Nearly 7% of tattooed and pierced students from both age groups reported complications, with differences in the prevalence of infectious and non-infectious effects higher in the first and second groups, respectively.

Gold and colleagues found that pierced youths had a perceived risk of complications that was significantly higher than the actual prevalence of complications [20]. In our study, although a majority of both samples were aware of health risks associated with body art practices, specific knowledge was scarce.

Adequate information given to tattooists and piercers can prevent health risks associated with body art. Simultaneously, public health education targeted at those who choose to get tattooed or pierced can increase patronage of licensed facilities and teach consumers what questions to ask about safe practice measures. Information on correct ways to take care of piercing and tattoo sites will also help avoid complications.

### Limitations of the study

As stated above, this study has some limitations. First, the two samples are not homogeneous in size or gender distribution. Second, schools and faculties were not randomly selected; we used convenience samples selected on the basis of teacher agreement, which could introduce selection bias. Third, the study considered only students, and therefore, the results cannot be generalized to the entire young population in the province of Naples.

Finally, we did not take students' social and economic conditions into account or determine if there is an association between body art practices and other maladaptive behaviors. As the aim of the study was focused on perceptions of health risks among youth, these factors are important [17]. Strengths of the study include the large size of the two samples, which can reduce the effects of confounding and bias. This study is also the first to compare knowledge about and perceptions of body art in two separate age groups.

## Conclusions

Although limited to students, this study involved a large sample of adolescents and young adults in the province of Naples. Findings indicate that body art practices are very widespread among secondary school and first-year university students. In comparisons between the two age groups, adolescents were not as well informed as university students, and more likely to seek out unauthorized operators. However, youth who knew about possible risks relied on piercers and tattooists as their primary sources of information. This finding underscores the need to use legislation to mandate health education of body art workers and equip them with the means to provide accurate health risk information to youths, who are particularly interested in body modification. In 2010, the Local Health Authority of the Campania Region released regional regulations that establish specific mandatory training on hygiene measures for tattooists and piercers.

Based on our findings, we also developed a booklet on body art and possible health risks associated with it. This has been distributed to the students from the high schools that participated in the study. In some of the schools, we have also met with students to discuss health topics related to body modification. Our goal in these efforts is to improve awareness of risks, and knowledge about how to effectively deal with them among the young Neapolitan population.

## Competing interests

The authors declare that they have no competing interests.

## Authors' contributions

GL, FG, MG and CM conceived and designed the study; defined the objectives, performed background analysis and bibliographic research; developed the questionnaire; and analyzed and interpreted data. They provided critical revisions for intellectual content and gave their final approval of the version to be published. CM, AV, EA, VDO, and RM distributed the questionnaire, acquired the data, and developed the database. They performed writing, and read and approved the manuscript. All authors read and approved the final manuscript.

## Pre-publication history

The pre-publication history for this paper can be accessed here:

http://www.biomedcentral.com/1471-2458/11/625/prepub

## Supplementary Material

Additional file 1 **Body art questionnaire**. An English version of the questionnaire administered to students. Click here for file
