# Supplementary material for: Awareness of health risks related to body art practices among youth in Naples, Italy: a descriptive convenience sample study
Source: BMC Public Health. 2011 Aug 5;11:625. doi: 10.1186/1471-2458-11-625 (PMC3223796; doi:10.1186/1471-2458-11-625)
Supplement: Additional file 1 — Body art questionnaire. An English version of the questionnaire administered to students. [file 1471-2458-11-625-S1.DOC]

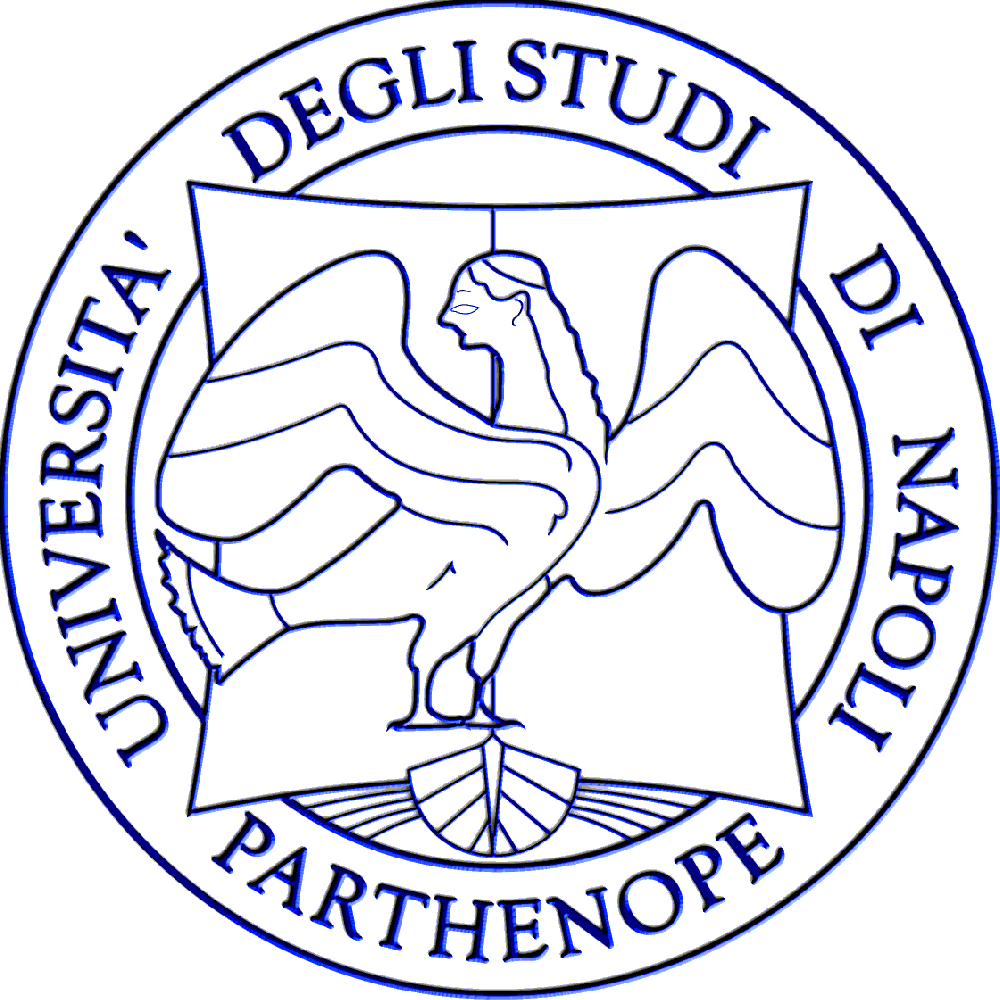


*Chair of Hygiene and Epidemiology,*

*University “Parthenope”of Naples*


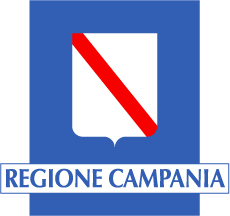


Local Health Authority of the Campania region

**EDUCATIONAL PROJECT FOR CUSTOMERS AND OPERATORS OF THE CAMPANIA REGION ABOUT THE KNOWLEDGE OF HEALTH RISKS ASSOCIATED WITH TATTOOING AND PIERCING**

The aim of the study was to evaluate the knowledge of the high school and university students about tattoo and piercing practices (**see endnote*). The questionnaire is anonymous. On the basis of the decree law n.196/2003 on the protection of personal information, the data analysis will be carried out in an aggregate way and the nominative results will not spread. If you accept the use of these information, return the questionnaire completed.

**MARK ANSWERS WITH X**

**Age** ⁯⁯ **Sex** M ⁯ F ⁯ **Place of residence** __________________________

**IN YOUR OPINION**

**There are any infectious diseases that can be transmitted through tattooing or piercing?** No ⁯ Yes Don’t know ⁯

**If they exist, what are among those listed here?**

⁯ Hepatitis B ⁯ AIDS ⁯ Gastritis ⁯ Hepatitis A ⁯ Hepatitis C ⁯ Tetanus ⁯

**Tattooing and piercing practices can cause non-infectious diseases?**

No ⁯ Yes Don’t know ⁯

**If they exist, what are among those listed here?**

⁯ Choking Oral cavity injuries ⁯ Bleeding Allergies ⁯ Abscess or cysts ⁯ Scars

Problems in the pronunciation

**Tattoos can be removed?** No ⁯ Yes Don’t know ⁯

**If yes, how?**

⁯ Surgical intervention ⁯ Ink aspiration ⁯ Other_______________________

**Piercing can be removed?** No ⁯ Yes Don’t know ⁯

**If yes, how?**

⁯ Surgical intervention ⁯ Spontaneous closing ⁯ Other_______________________

**Do you have a piercing?** No ⁯ Yes ⁯

⁯⁯

**At what age do you have practiced them (the first)?**  ⁯⁯ years

**If no, would you practice them?** No ⁯ Yes Don’t know ⁯

**Do you have a tattoo?** No ⁯ Yes ⁯

⁯⁯

**At what age do you have practiced them (the first)?**  ⁯⁯years

**If no, would you practice them?**  No ⁯ Yes Don’t know ⁯

**IF YOU HAVE A TATTOO OR A PIERCING, PLEASE ANSWER TO THESE QUESTIONS:**

⁯

**Have you practiced a tattoo/piercing because:**

It is fashionable Now you are more similar to your friends It betters your look

So you feel stronger ⁯ It can distinguish you from the others ⁯ Your preferred VIP have a tattoo/piercing ⁯ I don’t know

**Do you have informed your parents?** No ⁯ Yes ⁯

**Do you or your parents have signed an informed consent ?** No ⁯ Yes Don’t know ⁯

**Where do you have practiced the tattoo or the piercing?**⁯ At home ⁯ Street tattooist/piercer ⁯ Beautician Tattooist/pierced authorized facility ⁯

**Have you been informed about the health risks associated with these practices?**

No ⁯ Yes Don’t know ⁯

**If yes, how?**

⁯ Informed consent ⁯ By the operator ⁯ By another person/source

**The place was**:

Very dirty ⁯ Dirty ⁯ Clean⁯ Very clean ⁯

**Have the operator used sterile/disposable instruments?**

No ⁯ Yes Don’t know ⁯

**Do you have reported any complication?** No ⁯ Yes⁯

**If yes, what?**

⁯ Dermatitis ⁯ Allergies ⁯ Infections ⁯ Other _________________

**THANKS FOR YOUR COLLABORATION**

* To the aim of this study, tattoos are considered only if they involve the subcutaneous inoculation of coloured pigments; piercing represents the pierce of skin and mucous membranes (except the earlobe) with the insertion of rings or other metallic pieces.
